# CAM practitioners in the Australian health workforce: an underutilized resource

**DOI:** 10.1186/1472-6882-12-205

**Published:** 2012-11-02

**Authors:** Sandra Grace

**Affiliations:** 1 School of Health & Human Sciences, Room Z2.12, Southern Cross University, PO Box 157, Lismore, NSW, 2480, Australia

## Abstract

**Background:**

CAM practitioners are a valuable but underutilizes resource in Australian health care. Despite increasing public support for complementary and alternative medicine (CAM) little is known about the CAM workforce. Apart from the registered professions of chiropractic, osteopathy and Chinese medicine, accurate information about the number of CAM practitioners in the workforce has been difficult to obtain. It appears that many non-registered CAM practitioners, although highly qualified, are not working to their full capacity.

**Discussion:**

Increasing public endorsement of CAM stands in contrast to the negative attitude toward the CAM workforce by some members of the medical and other health professions and by government policy makers. The marginalisation of the CAM workforce is evident in prejudicial attitudes held by some members of the medical and other health professions and its exclusion from government policy making. Inconsistent educational standards has meant that non-registered CAM practitioners, including highly qualified and competent ones, are frequently overlooked. Legitimising their contribution to the health workforce could alleviate workforce shortages and provide opportunities for redesigned job roles and new multidisciplinary teams. Priorities for better utilisation of the CAM workforce include establishing a guaranteed minimum education standard for more CAM occupation groups through national registration, providing interprofessional education that includes CAM practitioners, developing courses to upgrade CAM practitioners' professional skills in areas of indentified need, and increasing support for CAM research.

**Summary:**

Marginalisation of the CAM workforce has disadvantaged those qualified and competent CAM practitioners who practise evidence-informed medicine on the basis of many years of university training. Legitimising and expanding the important contribution of CAM practitioners could alleviate projected health workforce shortages, particularly for the prevention and management of chronic health conditions and for health promotion.

## Background

CAM practitioners are a valuable but underutilized resource in Australian health care despite continuing public support for CAM as demonstrated by increased use of both CAM products and growth in the number of consultations with CAM practitioners. In South Australia between 1993 and 2004, for example, the number of survey respondents who had consulted a CAM practitioner rose from 20.3% to 26.5% [1-3]. In 2005 a national population-based survey found that 68.9% of respondents had used at least one of 17 CAM therapies in the previous 12 months and 64% had visited a CAM practitioner in the same period [4]. However, reports about CAM use often fail to specify whether CAM services were delivered by a member of the medical profession with CAM training or by a non-medically trained CAM practitioner. In fact discussions about the increasing prevalence and use of CAM rarely focus on the CAM workforce.

### The CAM workforce in Australia

#### Number of CAM practitioners

Accurate numbers of practitioners can only be given for nationally registered professions. The Australian Health Practitioner Regulation Agency was established in 2010 to implement a national registration and accreditation scheme across Australia [5]. In 2012 there were 560000 registered health practitioners in Australia including 4294 chiropractors, 1595 osteopaths, and 4000 Chinese medicine practitioners.

There have been few attempts to collect data on the number of non-registered CAM practitioners working in Australia. This is a difficult task because there is no central register across, or in many cases within, different CAM occupations and many CAM practitioners work part-time or casually and may not report such work as their occupation. Consequently, figures like those reported by the Australian Bureau of Statistics in 2001 (8533 CAM practitioners) [6] and by the Australian Government Productivity Commission in 2005 (8550 CAM practitioners representing 1.9% of all health occupations) [7] are likely to underestimate the size of the CAM workforce.

More accurate data may be obtained from professional associations. Most graduates of colleges and courses accredited with professional associations seek membership with their respective associations. Schedule 1 of the Therapeutic Goods Regulation listed 41 associations [8] but some estimates are much higher [9]. Table 1 was compiled using data from the professional associations identified in Hale’s [10] national survey of remedial therapists and Bensoussan et al.’s [11] national surveys of naturopathic and Western herbal medicine practitioners, and from three key associations which fell outside the ambit of their surveys. The combined membership of the Australian Traditional Medicine Society (ATMS) and the Australian Natural Therapists’ Association (ANTA), reportedly together representing 75% of the CAM workforce (excluding chiropractic and osteopathy), was approximately 12,000 in 2003 [12]. However, practitioners who have trained overseas, trained with a non-accredited college, who have not undertaken any formal training, or who have not sought professional association membership may also be in practice. It is therefore possible that the number of CAM practitioners in Australia is considerably higher than the official ATMS and ANTA figures. To further complicate matters, it is estimated that between 28% and 50% of non-registered CAM practitioners belong to more than one association [10,11,13]. Taking this into account, a more accurate estimate places the number of CAM practitioners in Australia in 2007 (who were not registered chiropractors, osteopaths or Chinese medicine practitioners, and who were members of CAM professional associations) at between 15447 and 22234.

**Table 1:** The Australian non-registered CAM workforce 2007

**CAM Professional Associations**	**No. of practitioners**
Australian Acupuncture & Chinese Medicine Assoc.	1600
Australian Association of Massage Therapists	6000
Australian Homoeopathic Association	400
Australian Hypnotherapy Association	275
Australian Kinesiology Association	421
Association of Massage Therapists	1232
Australian Natural Therapists Association	3900
Association of Remedial Masseurs	393
Australian Register of Homoeopaths	57
Australian Traditional Medicine Society	11571
Bowen Association	1202
Federation of Natural and Traditional Therapists including:	
Australian Association of Homotoxicology	163
Australian Naturopathic Practitioners Association	265
International Association of Trichologists	26
Reflexology Association of Australia	1000
International Federation of Aromatherapists	280
Massage Australia	500
National Herbalists’ Association of Australia	900
Psychotherapy & Counselling Federation of Australia	335
Shiatsu Therapists Association	374
**Total**	**30894**

Note: In 2007 there were 3924 nationally registered chiropractors, 1427 nationally registered osteopaths and 991 Chinese medicine practitioners registered in the state of Victoria.

Note: Three associations (Australian Ayurvedic Association, Queensland Association of Massage Therapists, and the Victorian Herbalists’ Association were not contactable by webpage or telephone). The Complementary Medicine Association did not disclose the number of members for privacy reasons.

#### Profile and work practices of CAM practitioners

The limited data available from the Chiropractic and Osteopathic Registration Boards suggests strong similarities between the professions: 61.5% of registered chiropractors and 67.6% of registered osteopaths are between 26 and 45 years of age; 35.6% of registered chiropractors and 31.3% of registered osteopaths are female; and the majority of practitioners practise in NSW and Victoria (60.2% of chiropractors and 80.2% of osteopaths) [14,15]. Chiropractic and osteopathic businesses operating at 30 June, 2010 generated an average income of $232,100 per business. Fee for service income earned per practitioner was $108,500 [16].

Little is known about the work practices of non-registered CAM practitioners. Three national surveys of CAM practitioners, funded by the Department of Health and Ageing, were conducted in Australia in 2000 [10,11,13]. These surveys were followed by a review of naturopathy and Western herbal medicine [17]. The picture of the CAM workforce (excluding the registered professions chiropractic and osteopathy) that emerged was of a predominantly female, urbanised workforce, with varying levels of training, and of relatively low income. It appears that most CAM therapists did not continue in full-time practice beyond five or six years. Table 2 presents some of the data from the three national workforce surveys described above. It appears that many CAM practitioners were underutilised in terms of their potential client reach and that the effects of statutory registration on the workforce may extend beyond conformity of training to enhancing the prospect of a secure and viable income.

**Table 2:** **Comparison of three national CAM workforce surveys**[9,10,12]

	**Remedial therapists**[9]	**Naturopaths, Western medical herbalists, acupuncturists**[12]	**Naturopaths, Western medical herbalists**[10]
**Average age (range)**	26-55 years	34-45 years	44 years
**Gender**	76%: female	74.8%: female	76%: female
**Practice location: metropolitan/rural**	54%: metropolitan	57%: metropolitan	Twice as many metropolitan as rural practices
**Type of practice**	64%: sole practitioner	59%: sole practices	30%: group practice
	18%: group practice	28%: group practice	
**Length of training (full time equivalent)**	41%: one year	42%: four years	6 months – 6 years
	37%: two year	(7.8%: bachelor degree)	
**Average number of consultations per week**	12	15.7	22.3
**Income**	91% less than	53% less than	
	$50 000 per annum	$30 000 per annum	
**Length of time in practice**	64%: 1–5 years	39%: 1–5 years	Average 6.7 years

Between 1996 and 2001 the growth in complementary health occupations represented the second highest increase (29.6%) of all health occupations after an unspecified group of health occupations [7]. This rapid increase in the CAM workforce appears to have had little effect on government planning and policy. For example, although the Australian Government Productivity Commission report acknowledged the growth of the CAM workforce, it did not address any role that CAM might play in the future health system. There was also no mention of CAM practitioners in the Federal, State and Territory governments’ $500 million Australian Better Health Initiative aimed at managing chronic illnesses and preventive medicine [18] despite these being the main reasons that people choose CAM [3,17,19,20].

The Australian Health Workforce Taskforce was established to manage major reforms to the Australian health workforce. Its agenda is to implement workforce reform and devise solutions that integrate workforce planning, policy and reform with the necessary reforms to education and training. Specific aims include increasing supply and reforming the workforce (e.g. by supporting new models of care, new and expanding roles, and multi-disciplinary teams). In this climate of significant health care reform it appears that little attention is being given to existing and potential contributions of CAM practitioners or to education reforms for the future, an area of neglect that is becoming increasingly entrenched.

The purpose of this paper is to discuss the unwillingness of the medical and other health care professions to recognize the actual and potential contribution of the CAM workforce to Australian health care and to propose strategies to enhance the quality of health care by recognising and embracing CAM practitioners.

## Discussion

### Why is the CAM workforce underutilized?

Increasing public endorsement of CAM stands in contrast to the negative attitude toward the CAM workforce by some members of the medical and other health professions and by government policy makers. There are long histories of territorial rivalries, power struggles, and, within current evidence-based medicine, disagreements over what constitutes legitimate evidence [21]. Marginalisation of CAM is evident in three key areas:

#### Prejudicial attitudes towards the CAM workforce

Failure to recognise the potential contribution of CAM to primary health care, and in some cases overt hostility to it, still exists within the medical profession [22,23]. A recent example is the Friends of Science in Medicine whose mission is to remove CAM courses from universities on the grounds that the pseudoscience they teach is not worthy of inclusion in higher education curricula [24].

Although some members of the medical and other health care professions have espoused a growing tolerance and endorsement of CAM [25-28], particularly since the emergence of a biomedical evidence-base for some CAM [29,30], the question of who is qualified to dispense and practise CAM remains problematical. In Australia, acupuncture, massage, meditation, hypnosis and spinal manipulation were the complementary therapies most commonly chosen by general medical practitioners for patient referral [31-33]. However, it is often unclear in such reports whether referral was for CAM services provided by medical practitioners who practised CAM or by non-medically trained CAM practitioners.

The Australian Medical Association’s Position Statement on complementary medicine recognised that scientific evidence-based aspects of complementary medicine are part of the repertoire of patient care and may have a role in conventional medical practice [34]. In the USA, 70–90% of family physicians who responded to a survey (n=180) considered such therapies as diet advice, exercise therapy, behavioural medicine, counselling and hypnotherapy to be legitimate medical practice [35]. Van Haselen [36] canvassed opinions of members of the medical profession and other health care workers on the integration of CAM into primary health care in the UK. Survey responses were received from 149 general medical practitioners, 24 nurses and 32 other primary care team members. Most respondents felt that only general medical practitioners or other non-CAM health professionals trained in CAM should provide CAM. Only 26% endorsed the provision of CAM by non-state registered practitioners. In one Australian study [37], medical practitioners who referred for CAM services preferred to refer to medical practitioners who practised CAM rather than to non-medically trained CAM practitioners.

#### Overlooking the CAM workforce in government policies

Government agencies and policy makers have an important influence on the practices of CAM practitioners through professional registration, course and training provider accreditation, regulation of the manufacture and sale of CAM products, and accreditation of providers for government insurance schemes. Government interest in CAM has escalated in response to the increasing public demand for CAM and the concomitant requirement to protect consumers of CAM [38]. Policies have enhanced the professional standing of some groups of CAM practitioners and compromised that of others. For example, government recognition through registration, Goods and Services Tax exemption, and inclusion in government insurance schemes went a long way to legitimising the role of some CAM practitioners in the health care system, but by omission reduced the standing of numerous others not so recognised.

Perhaps the most important recent change in government policy is the inclusion of CAM providers in the Australian government’s Medicare Benefits Schedule. Chiropractors and osteopaths have been included in allied health Medicare items for chronic disease management. These items enable general medical practitioners to plan and coordinate the health care of patients with chronic or terminal medical conditions, including patients with these conditions who require multidisciplinary, team-based care [39]. The plan entitles patients of allied health professionals contributing to this type of care to a government rebate. Importantly, this represents a major shift in government policy towards CAM, being the first time government funding has been allocated to any CAM services, albeit with medical oversight.

The Australian Government is also committed to E-Health to deliver safe, efficient and quality health care. According to the Australian Health Ministers’ Advisory Council [40] E-Health will allow health care providers to readily know who other providers are and where they are located to facilitate referrals and timely access to care. Any exclusion of qualified CAM practitioners from the national E-Health strategy will undoubtedly further marginalise them.

#### Exclusion from national registration

National registration guarantees a minimum educational standard. Little has changed since the Australian government’s Expert Committee on Complementary Medicines in the Health System [9] reported that “educational standards amongst Australian trained complementary medicine practitioners are extremely variable, and neither the public nor other healthcare practitioners have a reliable way of assessing who is sufficiently or appropriately qualified for safe, competent practice” (p. 24). Inconsistent educational standards for non-registered CAM practitioners are a major barrier to better utilization of these groups. The development of a national curriculum by the Australian National Training Authority in the Health Training Package HLT07 represents a genuine attempt by the Australian government to develop uniform curricula for some CAM occupations [41]. However, lack of resources to monitor compliance with training requirements has undermined the process. Other interest groups have emerged including the Australian Register of Naturopaths and Herbalists which aims to establish minimum standards of education in accordance with government requirements for the regulation of health practitioners [42].

Locating CAM courses in tertiary institutions has created enthusiasm for research to underpin many common CAM practices. While it may be erroneous to claim that all CAM courses are based on scientific evidence, it is probably fair to say that all CAM courses strive to be evidence-informed. Ernst [43] rightly points out that many claims about CAM’s efficacy are overstated. However, there is a risk that legitimate use of CAM, particularly for chronic disease management and for health promotion, might be overlooked, particularly in cases where a good evidence base is emerging.

### Taking the CAM workforce into the fold

Potential advantages of better utilisation of the existing and future CAM workforce include:

•Drawing on an underutilized resource to alleviate workforce shortages in the health care sector [44-46]. Pressures on general medical practitioners to manage the increasing incidence of chronic illness and to introduce more proactive health promotion strategies may provide a strong impetus for collaboration with CAM practitioners. Educational opportunities could be provided for CAM practitioners to upgrade their skills in their own disciplines and in identified areas of shortage and predicted need.

•An expanded patient-centred and holistic approach to health care, which is central to the way CAM is practised [47]. The most widely discussed benefits of integrating CAM and conventional medicine relate to its patient-centred, holistic, health-focused approach, as well as its wide range of treatment options for patients with chronic health conditions and for those wanting to prevent illness and to promote their health.

•Potential positive interactions between CAM and conventional medicine. There has been much focus in the literature and the media on the potentially hazardous interactions of CAM medications and pharmaceutical products. However, it is worth noting that positive aspects of combining some CAM medications with prescribed pharmaceuticals have also emerged. Examples include co-prescribing antibiotics and lactobacillus acidophilus to replace the intestinal bacteria destroyed by antibiotics, and using milk thistle (Silybum marianum) for the prevention of liver dysfunction in clients undergoing anticancer treatment [48].

•New models of delivery. The literature presents various ways in which CAM and conventional medicine can be integrated and it appears that strategies can be successfully developed to facilitate integration. Two dominant models of integration are described in the literature:

(1) Selective fusion of the most effective elements of both CAM and conventional medicine based on health outcomes.This is the ideal model described by Lewith and Bensoussan [49]. Both biomedical evidence and clinical efficacy are valued. CAM and conventional medicine are complementary to each other and CAM practitioners and members of the medical profession are co-workers with equal input and standing. The Birkenholm Centre in Denmark was established as such a model [50] as was the Marylebone Health Centre in London in which condition-based guidelines (as opposed to individualised care plans) were used for CAM service delivery [51].

(2) Selective incorporation of some elements of CAM into conventional medicine. This model may involve initially directing patients to members of the medical profession for conventional medical assessment. If referral to a CAM practitioner is required it is carried out under the aegis of medical practitioners [52-57]. The Australian Government’s Medicare rebate system exemplifies this model in relation to subsidising chiropractic, podiatry, psychology and other allied health treatments for patients with chronic diseases. Subsidies are available only if the services are part of enhanced care plans and are supervised by general medical practitioners [58].

Models of care where medical practitioners are gatekeepers of patient care rely in part on the knowledge and attitudes of the referring medical practitioners to establish health care teams. Educating medical practitioners about CAM has been advocated by the Australian Medical Association [34], as it has by the US Institute of Medicine’s Academy of Science Committee on the Use of CAM [59] and by similar bodies in other countries, as a vital strategy to enable competent advice about CAM to be given by medical practitioners and to ensure competent referrals. CAM education for medical practitioners has been poorly endorsed in Australian medical education [60]. Collaborative models of health care delivery may be encouraged by interprofessional learning strategies which could foster such competencies in learners as teamwork, leadership, and the ability to identify shared goals in patient care [61,62], especially when it is introduced in the early years of training [63,64]. Logistical and resource constraints, including the paucity of appropriately skilled educators, have been identified as inhibiting the implementation of interprofessional learning, as have diverse learning styles and levels of motivation in students [65,66]. In Australia, examples of interprofessional education are isolated. It appears that it currently exists only on the margins of health professional curricula and practice.

•Patient disclosure. Patients often fail to disclose their CAM use to their general medical practitioners [4]. Recognition and inclusion of the CAM workforce in government initiatives like E-health would facilitate comprehensive and accurate records of the totality of patient health care. Moreover, such recognition and inclusion could encourage patients to disclose their use of CAM products and services to their general medical practitioners.

•Cost-benefit. In Australia most CAM services are provided at the patient’s expense and arguments for government subsidy turns on cost-benefit analysis. In Australia, as elsewhere, there has been some optimism that the use of CAM with its focus on low cost lifestyle management and health maintenance might reduce future medical costs [67]. It is clear that there is a need for rigorous research in this area and that there are methodological difficulties to be overcome [68,69].

Studies of the costs of specific CAM occupations for specific conditions compared to conventional medical treatment have shown inconsistent results [70]. Using cost per quality adjusted life years, additional costs of CAM care have been shown to be wholly or partly offset by reductions in the cost of conventional care [71]. Systematic reviews by White and Ernst [72] and Canter, Coon and Ernst [68] showed that spinal manipulation and acupuncture actually increased the costs to clients when compared to other treatments approved for use by the National Health Service in the UK, although in estimates of cost per quality adjusted life year they compared favourably. In 1998, the Swiss government commissioned an extensive study of CAM treatments including homoeopathy, traditional Chinese medicine, herbal medicine and anthroposophic medicine to determine if they were effective and cost-effective. The results of the study were responsible for the reinstatement of the Swiss government's health insurance program to support CAM [73,74]. Sarnat and Winterstein [75] in the US found promising clinical and cost saving results when they investigated primary care physicians who integrated non-pharmaceutical/non-surgical approaches with allopathic medicine. Cost-effectiveness of this sort strengthens the case for the inclusion of CAM services in government-subsidised programs.

### Potential disadvantages of greater utilisation of the existing and future CAM workforce include

•Delayed diagnosis and therapy. Greater utilisation of CAM practitioners by government agencies and medical and other health practitioners could promote CAM practitioners’ primary contact role in health care. Concerns have been raised about the competence of some CAM practitioners to adequately fulfil this role [76]. For patients, this could mean an incorrect or delayed diagnosis, delayed therapy or absence of adequate conventional therapy, less than optimal health outcomes, and a waste of money and time.

•The most likely model of care which integrates medical and CAM practitioners to be taken up places the general medical practitioner as the gatekeeper for patient care. For CAM practitioners this represents a loss of autonomy and potentially a compromise of their treatment approach [76]. Shuval and Mizrachi [77] found that CAM gained legitimacy when they worked collaboratively in practices with general medical practitioners but that they did not have the same status as other health care providers in these practices. That CAM runs the risk of being subsumed under conventional medicine is borne out by the experience of several clinics where CAM and conventional medicine practitioners practise together [30,78,79].

### Strategies for making better use of the CAM workforce

The following strategies need to be prioritised if the potential benefits of better utilization of the CAM workforce are to be realized:

•National registration: it appears that guaranteed minimum standards of education of all CAM practitioners can only be achieved by national registration. The Australian Health Practitioners Regulation Agency could review further CAM occupations for registration like naturopathy and Western herbal medicine which continue to develop a strong evidence-base. Non-registered CAM occupations are divided over national registration: some cite fears about government intervention and bureaucratic restrictions of practice; others make calls for guaranteed minimum education requirements, increased standing in the community and eligibility for government funded schemes.

•Interprofessional education: embedding interprofessional educational and practice capabilities in all health professional curricula could encourage even-handed examination of the contribution of CAM practitioners to patient health care, referral and co-management.

•CAM E-Health: it is imperative that registered and other suitably qualified CAM practitioners be included in E-Health initiatives to facilitate referral from other health practitioners, and to enable a range of research opportunities, including research into cost-effectiveness and interactions between CAM and conventional medicine.

•Re-designing roles of CAM practitioners: Professions have always responded to new ways of perceiving health and illness, new technologies, innovations in education and new regulation, and have always had changing roles and status in society [80]. Professional boundaries are dynamic and change according to such pressures as workforce shortages, performance-based management principles and consumer preference. According to Nancarrow and Borthwick [80], a workforce can change within a single discipline through diversification and specialisation, and/or can undergo vertical and horizontal substitution, which occur when a discipline moves outside its traditional boundaries, There are opportunities to review current roles of CAM practitioners in the workforce with a view to re-designing roles, including performance in multidisciplinary teams (diversification and specialisation), especially in identified areas of need (e.g. prevention, treatment and management of chronic conditions, obesity, rural and remote community health). The CAM practitioner role could be legislated in a similar fashion to that of nurse practitioners who conduct autonomous and collaborative nursing practice in an advanced and extended clinical role (vertical substitution). Nurse practitioners’ tasks include prescribing medication, ordering diagnostic investigations, and referral to other health care practitioners. CAM practitioners already provide a valuable service in both community and private settings. Their particular strengths would be their focus on lifestyle medicine, health promotion and chronic disease management using simple, non-invasive and often inexpensive treatment approaches. Ordering diagnostic investigations could transform the practices of many CAM practitioners who currently depend on medical practitioners who may not support the rationale for their requests. However, it is likely that there would be legislative barriers and pockets of strong opposition from other health professions to overcome, as there have been for nurse practitioners [81]. Such opposition might raise objections concerning over-prescribing and over-diagnosing.

•Promoting CAM research: funding research that investigates the productivity and performance of CAM practitioners and multidisciplinary teams that include CAM practitioners is urgently required to develop a strong evidence base for CAM practice and to build research infrastructure and capacity among CAM academics and practitioners, Fostering collaborations with international groups like CAMbrella, a network of European research institutes in CAM, could contribute to our understanding of the current status of CAM in Australia and promote future research activities [82].

•Other collaborations: Other innovative strategies include developing a role for CAM practitioners in Australia’s Medicare Local health reform initiative. Medicare Local is a nation-wide network of primary health care organisations established to coordinate primary health care delivery directed to local care needs and service gaps. They will drive improvements in primary health care and ensure that services are better tailored to meet the needs of local communities. One of their key roles involves supporting local primary care providers, such as general medical practitioners, practice nurses and allied health providers, to adopt and meet quality standards. Collaborations could also be developed in Australia’s General Practitioner Super Clinic program, which is establishing multidisciplinary primary care health services and education and training placements in multidisciplinary care settings and also targets the health needs and priorities of their local community.

•Further CAM education programs for medical practitioners. Australian universities have been slow to embrace CAM education programs for medical practitioners, unlike their counterparts in the UK, Europe, US and Cuba. In the Cuban model medical practitioners are trained to practise CAM themselves [83]. It is likely that the reach of most Australian programs will be to educate medical practitioners to be informed advisers, not to practise CAM.

## Conclusion

Some sectors of the CAM workforce are largely ignored despite their growing participation in clinical care. The greatest barriers to recognising the contribution of CAM practitioners are its limited biomedical evidence base and concerns over the competence of CAM practitioners. It would appear that while there is evidence that positive attitudes to CAM therapies among members of the medical profession and other health professionals are increasing, the same measure of acceptance does not appear to apply to CAM practitioners. Members of the medical profession in particular may be more ready to use CAM therapies, or refer patients to other medical practitioners who practise CAM, than to acknowledge a basis of equality for CAM practitioners. In this climate of uncertainty unregistered CAM practitioners are often marginalised and their important potential contribution to patient care overlooked.

Better utilisation of the CAM workforce, in particular of those members with sound educational qualifications (e.g. naturopaths, herbalists and nutritionists graduating with Bachelors degrees from universities) could address some specific aims of the Australian Health Workforce Taskforce, including increasing supply and reforming the workforce. Priorities for better utilisation of the CAM workforce include: (1) increased opportunities for national registration, (2) inclusion in interprofessional education and practice, (3) inclusion in the government’s E-Health initiative, (4) developing new and expanded roles for CAM practitioners and multi-disciplinary teams which include CAM practitioners, and (5) promoting CAM research. The Australian public is increasingly integrating its conventional medical care with CAM care, particularly those patients with chronic and complex care needs and those wanting to promote their health. The time has arrived to legitimise and expand the important contribution of CAM practitioners to the Australian health care system.

## Summary

Despite increasing public support for CAM it appears that many non-registered CAM practitioners, although highly qualified, are not working to their full capacity. The greatest barriers to recognising their contribution are the limited biomedical evidence base for some CAM and concerns over the competence of CAM practitioners. Priorities for better utilisation of the CAM workforce include establishing a guaranteed minimum education standard for more CAM occupation groups through national registration, providing interprofessional education that includes CAM practitioners, developing courses to upgrade CAM practitioners’ professional skills in areas of identified need, and increasing support for CAM research. Legitimising and expanding the important contribution of CAM practitioners could alleviate projected health workforce shortages, particularly for the prevention and management of chronic health conditions and for health promotion, and provide opportunities for redesigned job roles and new multidisciplinary teams.

## Competing interests

The author declares that they have no competing interests.

## Authors’ contribution

SG is solely responsible for the intellectual content of this article and for drafting and revising the manuscript.

## Pre-publication history

The pre-publication history for this paper can be accessed here:

http://www.biomedcentral.com/1472-6882/12/205/prepub
